# Plasma Cytokine and Caspase-1p20 Profiles in Pre-Pandemic and Long COVID-Associated Postural Orthostatic Tachycardia Syndrome

**DOI:** 10.3390/biomedicines14071605

**Published:** 2026-07-17

**Authors:** William T. Gunning, John W. Spatafore, Michael P. Morran, Beverly L. Karabin, Benjamin R. Hart, Blair P. Grubb

**Affiliations:** 1 Department of Pathology, University of Toledo Medical Center, Toledo, OH 43614, USA; john.spatafore@rockets.utoledo.edu; 2 Department of Surgery, University of Toledo Medical Center, Toledo, OH 43614, USA; michael.morran@utoledo.edu; 3 Department of Medicine, University of Toledo Medical Center, Toledo, OH 43614, USA; beverly.karabin@rockets.utoledo.edu (B.L.K.); benjamin.hart@utoledo.edu (B.R.H.); blair.grubb@utoledo.edu (B.P.G.)

**Keywords:** POTS, long COVID, PACS, NLRP3 inflammasome, Caspase1, innate immune system cytokines

## Abstract

**Background:** Prior to the COVID-19 pandemic, the etiology of postural orthostatic tachycardia syndrome (POTS) remained elusive. Since the pandemic, a newly recognized disorder, termed Long COVID, has emerged with a significant subset of patients developing dysautonomia and a multitude of comorbidities consistent with POTS. **Aim:** The aim of this study was to determine if pre-pandemic POTS and Long COVID POTS share a common inflammatory-associated biomarker profile. **Methods:** Volunteers were recruited for four study groups; patients diagnosed with POTS prior to the pandemic, Long COVID-associated POTS, SARS-CoV-2-recovered controls, and naïve controls. All participants completed a COMPASS-31 survey and a medical history questionnaire. Plasma biomarkers of the innate and adaptive immune system were quantified using a custom multiplex bead assay and ELISAs. **Results:** Both POTS cohorts demonstrated indistinguishable and significant elevations in 14 of the 15 measured biomarkers including markers of the NLRP3 axis (Caspase-1p20, interleukins IL-1β, IL-18), regulatory cytokine IL-10, and immune activation markers (sCD40L, sCD40, sCD30) compared to controls. Multivariate PERMANOVA analysis revealed no significant difference in global cytokine profiles between the two POTS cohorts. Random Forest classification accurately distinguished POTS from controls, with IL-18 emerging as the most important feature. **Conclusions:** These associative findings suggest that pre-pandemic POTS and Long COVID-associated POTS share a distinct inflammatory profile among measured cytokines. The identification of IL-18 as a key biomarker, alongside Caspase-1p20 and other inflammatory cytokines, are compatible with inflammasome-related signaling. Further investigation is necessary to characterize the role of the inflammasome, platelet activation, and immune dysregulation in POTS and Long COVID.

## 1. Introduction

Severe SARS-CoV-2 infection induces a dysregulated immune response, often termed a cytokine storm, that drives significant thromboembolic complications and acute respiratory distress syndrome (ARDS). These outcomes are especially common in patients with cardiovascular risk factors including obesity and diabetes [1,2]. This hyperinflammatory state results from innate immune signaling and macro- and microvascular immunothrombosis, which involves platelet activation [3]. Activation of the innate immune system involves the assembly and oligomerization of the nucleotide-binding oligomerization domain (NOD)-like receptor protein 3 (NLRP3) inflammasome, a macromolecular complex that facilitates proteolytic cleavage of pro-inflammatory precursors [4,5,6]. While primarily characterized within the cytoplasm of leukocytes and platelets, NLRP3 expression has also been reported in epithelial cells, endothelial cells, adipocytes, neurons, and glial cells.

Upon stimulation, NLRP3 recruits the adaptor molecule ASC (apoptosis-associated speck-like protein containing a CARD (caspase recruitment domain)) and pro-caspase-1 to assemble the inflammasome complex [7]. Once assembled, pro-caspase-1 undergoes auto-catalytic activation to generate Caspase-1, which subsequently proteolytically cleaves pro-IL1β and pro-IL-18 into active pro-inflammatory cytokines. Recent reports have implicated the NLRP3 inflammasome and innate immune cytokines in the severity of SARS-CoV-2 infections [8,9]. Biomarkers of an activated NLRP3 inflammasome include elevations of Caspase-1p20, IL-1β, and IL-18 in plasma [10].

Persistent symptoms following SARS-CoV-2 infection characterize post-acute COVID-19 syndrome (PACS), commonly referred to as Long COVID. Clinical studies indicate an estimated 66–79% of patients with Long COVID exhibit symptoms of dysautonomia, with a substantial proportion meeting the diagnostic criteria of postural orthostatic tachycardia syndrome (POTS) [11,12,13]. Reflecting this trend, a recent large-scale population-based study comparing pre- and post-pandemic health records demonstrated a 14-fold increase in the incidence of POTS [14].

While the pathogenesis of POTS remains incompletely understood, the current literature has documented the presence of circulating autoantibodies in affected patients [15,16]. Clinically, POTS is defined by symptoms of orthostatic intolerance lasting at least three months, accompanied by an increased heart rate of greater than or equal to 30 beats per minute (bpm) from a supine baseline, or a rate that exceeds 120 bpm within the first 10 min of standing or on an upright tilt table, in the absence of significant orthostatic hypotension [17,18,19]. This abnormal physiological state is driven by inadequate vascular peripheral resistance in response to orthostatic stress resulting in excessive distal venous pooling of blood in the peripheral areas of the body [20,21,22]. The resultant effective decline in circulatory volume triggers a compensatory baroreflex-mediated increase in heart rate and myocardial contractility. Progressive venous pooling can overwhelm this compensation leading to varying degrees of cerebral hypoperfusion and symptoms of orthostatic intolerance.

The diagnostic challenge of POTS is reflected by an average diagnostic delay of five years, driven by a constellation of disparate clinical symptoms including palpitations, Raynaud’s phenomenon, cognitive impairment (‘brain fog’), irritable bowel syndrome, hypermobility (Ehlers–Danlos syndrome in a third of the cases), and bleeding symptoms [23]. Among proposed etiologies for POTS, Epstein–Barr virus and other pathogens have been identified as potential initiating events; frequently, patients report a history of mononucleosis prior to development of debilitating symptoms [24]. We previously identified elevated titers of autoantibodies against the alpha-1 adrenergic and M4 muscarinic acetylcholine receptors in a cohort of POTS patients, [25]. To further characterize immune dysregulation, we then identified a significant elevation in specific innate immune system cytokines, including IL-1β and IL-18 [26].

The current study aimed to perform a comparative analysis of inflammatory biomarkers in patients diagnosed with POTS prior to the pandemic versus patients who developed POTS following SARS-CoV-2 infection. Our working hypothesis was that these groups would demonstrate similar cytokine signatures distinct from controls, suggesting a shared inflammatory profile associated with post-viral dysautonomia.

## 2. Materials and Methods

### 2.1. Sex as a Biological Variable

Our study included females and male participants; similar findings are reported for both sexes. While both POTS and Long COVID exhibit a strong female predominance, our study groups were demographically representative of patients diagnosed with POTS. Accordingly, sex was evaluated as a covariate in statistical analyses.

### 2.2. Patients

Volunteers were recruited across four defined cohorts: Group (1) healthy controls who had no history of SARS-CoV-2 infection (*n* = 46), Group (2) patients who had been diagnosed with POTS prior to the pandemic (n = 70), Group (3) healthy controls who had a history of SARS-CoV-2 infection and recovered (recovered controls, n = 53), and Group (4) patients who developed Long COVID, then subsequently diagnosed with POTS (n = 67). All POTS diagnoses (Groups 2 and 4) were established at our Syncope and Autonomic Disorders Clinic. Inclusion required a minimum six-month history of orthostatic intolerance manifested by orthostatic tachycardia, weakness, light-headedness, fatigue, and pre-syncope. Diagnosis was based upon clinical history, physical examination, and head-upright tilt table analysis in the fasting state. Patients in Group 2 were diagnosed prior to the pandemic and patients in Group 4 required symptom onset following a documented positive SARS-CoV-2 screening test.

**Figure 1:**
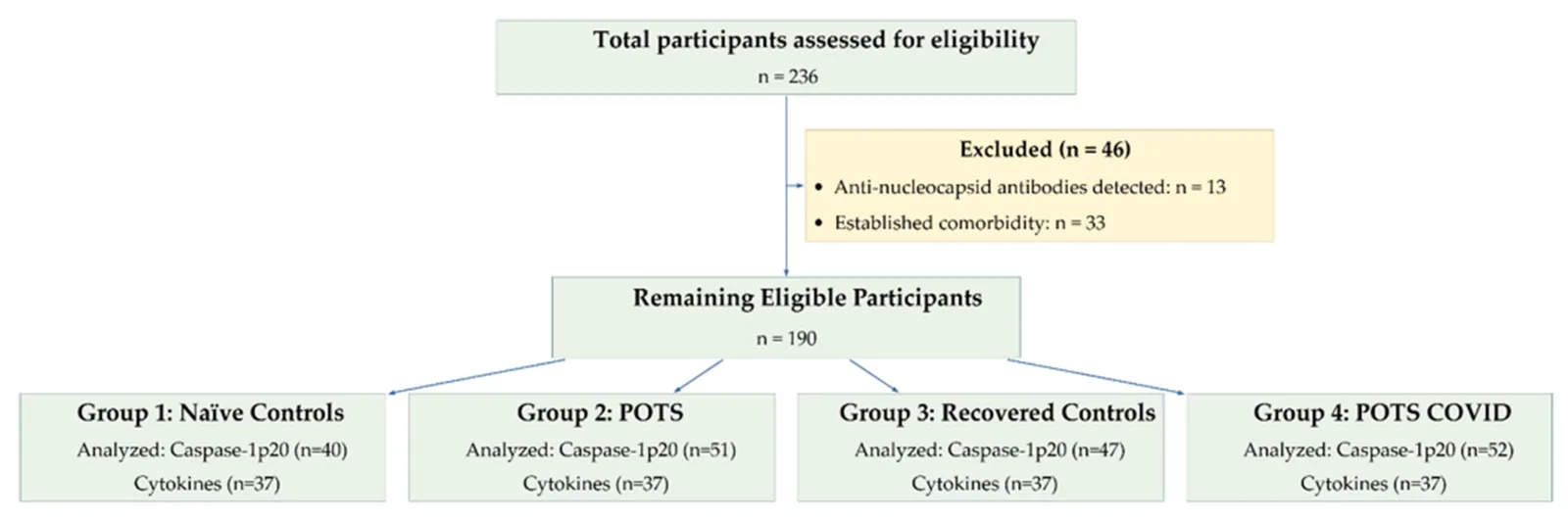
Enrollment and group allocation flow diagram. This flow diagram outlines the inclusion criteria and group enrollment progression. A total of 236 participants were assessed for eligibility, of whom 13 were excluded due to a positive anti-nucleocapsid antibody test and 33 were excluded for an established comorbidity. The remaining 190 participants composed of four groups: Naïve controls (n = 40), POTS (n = 51), recovered controls (n = 47), and POTS COVID (n = 52).

Each volunteer completed questionnaires detailing medical history, approximate date of COVID-19 infection or vaccination, and the Composite Autonomic Symptom Score-31 (COMPASS-31) [27,28,29]. COMPASS-31 is a validated 31-question instrument that evaluates multi-system autonomic dysfunction utilizing weighted scores. Peripheral blood samples were obtained from all participants to quantify plasma concentrations of cytokines and chemokines. Samples were also screened for SARS-CoV-2 antibodies using enzyme-linked immunosorbent assay (ELISA) kits targeting both viral nucleocapsid and spike proteins specific to the virus. Individuals were excluded from Groups 1 and 2 if anti-nucleocapsid antibodies to the SARS-CoV-2 virus were detected. Individuals that reported multi-system diseases of any etiology or established autoimmune disorders (i.e., Hashimoto’s thyroiditis) were excluded from all groups (Figure 1).

### 2.3. Cytokines and Chemokines

To evaluate activation of the innate immune system, plasma concentrations of Caspase1-p20, the active enzymatic subunit of Caspase-1, were quantified for all eligible participants utilizing ELISA kits (R&D Systems; Cat #DCA100, Minneapolis, MN, USA). For all other cytokine and chemokine assays, a balanced subset of 37 participants was randomly selected from each cohort (Figure 1). All samples were tested in duplicate. A panel of 14 pro-inflammatory cytokines were included, based upon a previous study of identified immune cytokines in POTS patients [26]. This panel consisted of IL-1β, IL-6, IL-8, IL-10, IL-17, IL-18, IL-21, IFNβ, IFNγ, TNFα, sCD30, sCD40, sCD40L (CD154), and MCP-1 (chemokine ligand 2/CCL2). Biomarkers were quantified utilizing a custom RayPlex^®^ Human Multiplex Bead Array as utilized in our previous study to assess 14 cytokine/chemokine biomarkers from RayBiotech, Inc. (Peachtree Corners, GA, USA). The multiplex bead system for flow cytometry enabled simultaneous quantification of the multiple analytes. All marker concentrations were quantified by reference to analyte-specific standard curves. All analyses were performed by RayBiotech in Peachtree Corners, GA, USA.

### 2.4. Statistical Methods

Statistical analysis was conducted using R statistical software (v4.5.1: R Core Team, 2025) and associated packages including ggplot2, vegan, caret, Random Forest, pROC, and glmnet [30,31,32,33,34]. Data was graphed using SigmaPlot 16.0 (Grafiti, LLC, Palo Alto, CA, USA). Continuous variables were assessed for normality (Shapiro–Wilk test) and homogeneity of variance (Levene’s test). Data are presented as mean [standard deviation] or median [interquartile range]. Baseline comparisons utilized one-way analysis of variance (ANOVA) or the Kruskal–Wallis test with Benjamini–Hochberg (BH) adjustment, with post hoc comparisons using Dunn’s test. Categorical variables are reported as percentages and compared with Fisher’s exact test. Across all analyses, a *p*-value of less than 0.05 was considered statistically significant.

To approximate a normal distribution for subsequent analyses, concentrations of cytokines were log2-transformed. Prior to transformation, zero values were assigned one half the minimum concentration detected across all samples. To account for the known female predominance in POTS, a targeted sensitivity analysis was performed restricting the cohort to female participants, with biomarker differences evaluated using the Kruskal–Wallis test with BH adjustment. Additionally, multiple linear regression (MLR) models were constructed to assess the independent effect of POTS status on each biomarker for the full cohort, adjusted for biological sex.

Global cytokine profiles differences were assessed using Permutational Multivariate Analysis of Variance (PERMANOVA), based on Euclidean distance with 9999 permutations. Beta dispersion analysis was employed to test the assumption of homogeneity of multivariate dispersion. Following a significant global result, pairwise PERMANOVA comparisons were conducted between groups with Holm-adjusted *p*-values.

Multivariate and machine learning analyses were implemented to further characterize the cytokine profiles of the cohorts. Preliminary analysis found no significant difference between the respective POTS and Control subgroups (PERMANOVA, *p* > 0.0). Therefore, for these analyses, the subgroups were collapsed into a single POTS cohort and a single Control cohort.

A robust panel of cytokines was identified using a Random Forest-based Recursive Feature Elimination (RFE) analysis with 10-fold cross validation repeated over 100 random seeds. The final biomarker panel was defined by cytokines consistently selected (>75%) across the 100 iterations.

A Random Forest classifier adjusted for age, weight, and sex evaluated the predictive power of the selected biomarker panel. Model performance was assessed using 10-fold cross-validation repeated 100 times and quantified by the area under the receiver operating characteristic curve (AUC-ROC). Relative contributions of each variable to model performance were ranked using permutation-based feature importance scores. Univariate AUC-ROCs were calculated for the top three variables within the panel.

The biomarker panel’s ability to predict clinical severity (COMPASS-31 score) in the POTS cohort was evaluated utilizing a Gamma-distributed, log-link generalized linear model (GLM) adjusted for confounders. Model features were pre-selected using cross-validated. Least Absolute Shrinkage and Selection Operator (LASSO) regression across 100 random seeds. The final biomarker panel for regression was composed of the cytokines demonstrating high selection frequency (>75%) across the iterations and its overall significance was assessed using an F-test.

### 2.5. Study Approval

Our prospective case–control study was approved by the Institutional Review Board of The University of Toledo Medical Center (UTIRB#301105). We conducted the study beginning in July 2021 through June 2023. All participating subjects provided informed consent.

### 2.6. Artificial Intelligence

Artificial intelligence (GenAI) was not used in this paper to generate text, data, or graphics, or to assist in study design, data collection, analysis, or interpretation.

## 3. Results

### 3.1. Demographics

**Figure 2:**
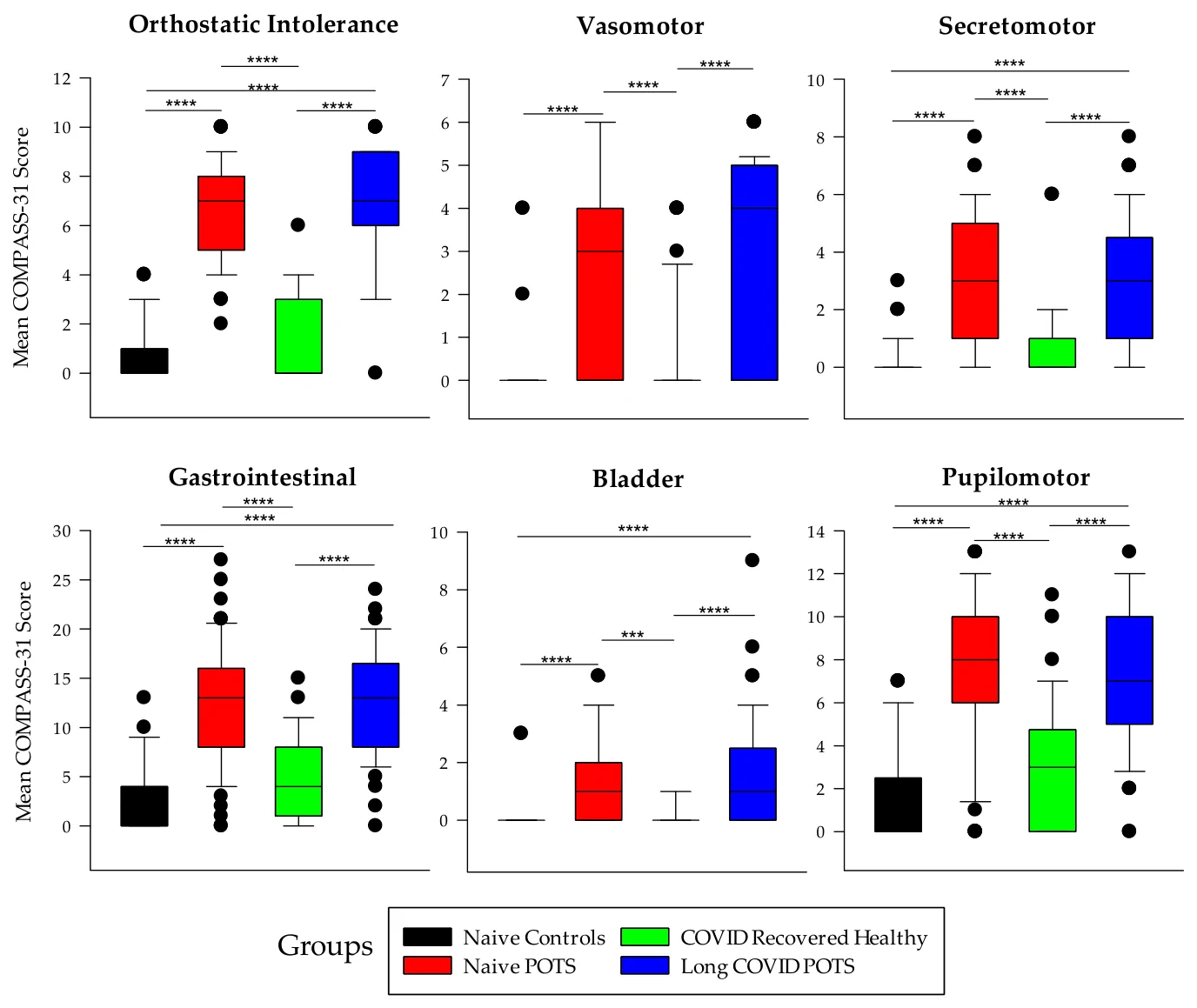
COMPASS-31 Autonomic Symptom Domain Scores Among Study Cohorts. Box plots present the median and interquartile ranges for the scores of orthostatism, vasomotor, secretomotor, gastrointestinal, bladder, and pupillomotor domains in POTS, POTS COVID, and control cohorts. Horizontal brackets indicate significant pairwise differences between cohorts (adjusted post hoc significance: *** *p* < 0.001, **** *p* < 0.0001).

**Figure 3:**
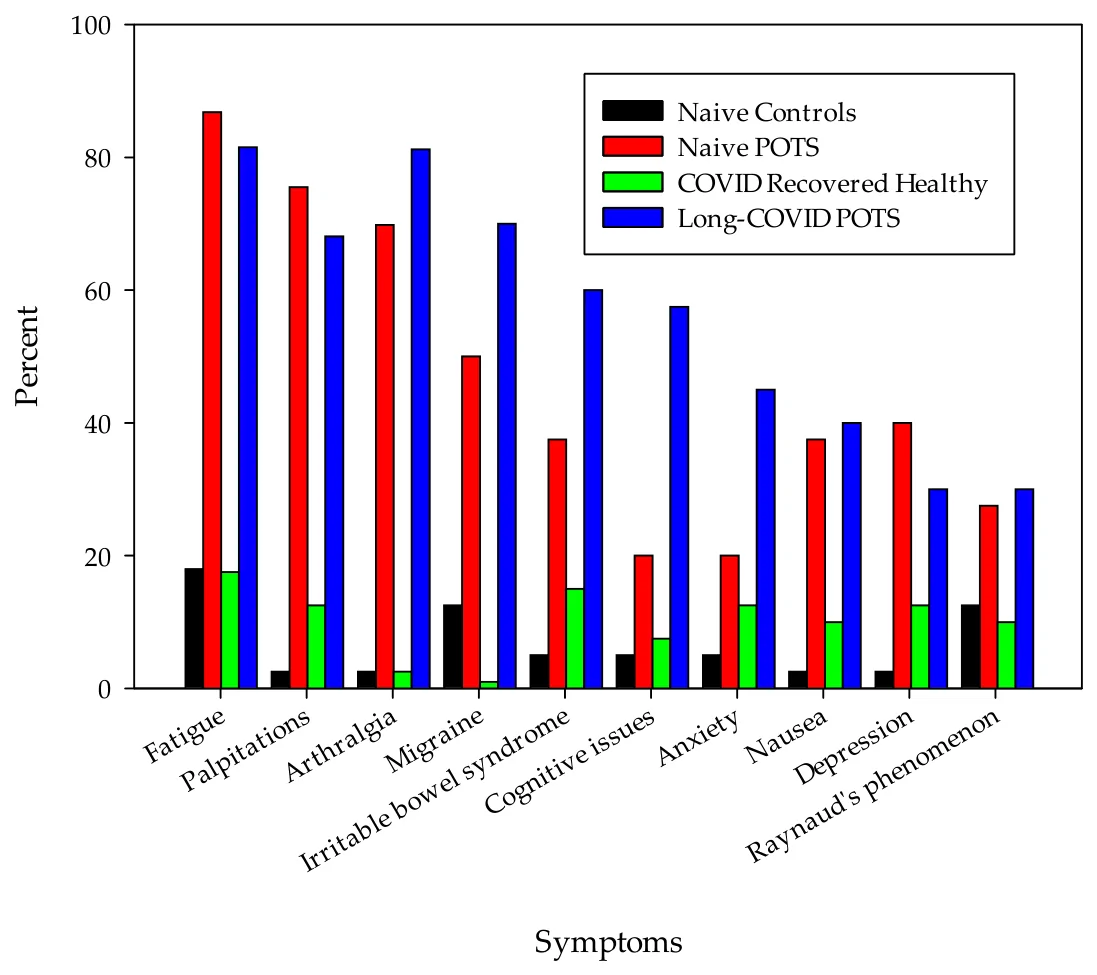
Comparison of common symptoms in study groups. Common symptoms reported by study groups consistently demonstrate POTS cohorts have a myriad of multiple comorbidities compared to controls. These symptom frequencies are consistent with the current literature for POTS patients and are often the basis of difficulty in diagnosing POTS.

A total of 236 volunteers were initially assessed for eligibility (Figure 1). Following screening, 46 individuals were excluded due to the detection of anti-nucleocapsid autoan-tibodies (n = 13) or the presence of established comorbidities (n = 33). The remaining 190 eligible participants were stratified into four cohorts: naïve controls (n = 40), POTS (n = 51), recovered controls (n = 47), and POTS COVID (n = 52). The study cohorts consisted predominately of young females (range: 62–96%) of comparable ages (*p* = 0.3, Table 1). COMPASS-31 scores, which assess severity of dysautonomia, differ significantly between the control and POTS cohorts (Figure 2). Both control cohorts scored below the established cutoff value of 20, whereas both POTS cohorts exhibited scores consistent with significant dysautonomia (POTS Naïve 30.7 ± 1.9 (SE) and Long COVID POTS 34.1 ± 1.8 (SE)) consistent with significant dysautonomia (Table 1). Figure 2 demonstrates orthostatic intolerance as the most significant COMPASS 31 symptom category for our POTS patients, followed by gastrointestinal issues and pupillomotor issues. The POTS and POTS COVID cohorts demonstrated similar symptom profiles (Figure 3), the most prevalent of which being fatigue (86.8% and 81.2%), palpitations (75.5% and 68.1%), and arthralgia (69.8% and 81.2%). However, depression was reported more frequently in the naïve POTS cohort.

### 3.2. Cytokines and Chemokines

In univariate analysis, Caspase1p20 and 13 of the 14 investigated cytokines were significantly elevated in the POTS and POTS COVID subgroups compared to controls (Table 2, Supplementary Figure S1). No significant differences were observed between the two POTS subgroups or between the naïve control and recovered control subgroups. In a targeted female-only sensitivity analysis, all previously significant biomarkers retained their statistical significance (Supplementary Table S1). Furthermore, MLR modeling confirmed that POTS status remained a robust, independent predictor of these 14 biomarkers across the full cohort after adjusting for biological sex (Supplementary Table S2).

A global assessment of the 14-cytokine panel revealed a significant difference between groups (PERMANOVA: R^2^ = 0.102, *p* < 0.001), with no significant dispersion differences between groups (*p* = 0.707). Post hoc comparisons confirmed significant differences between the POTS and control subgroups. However, no differences were found between POTS and POTS COVID subgroups (*p* = 0.774), nor between control subgroups (*p* = 0.638). Consequently, the two POTS subgroups were combined into a single POTS cohort, and the two control subgroups were combined into a single control cohort for subsequent analysis.

**Figure 4:**
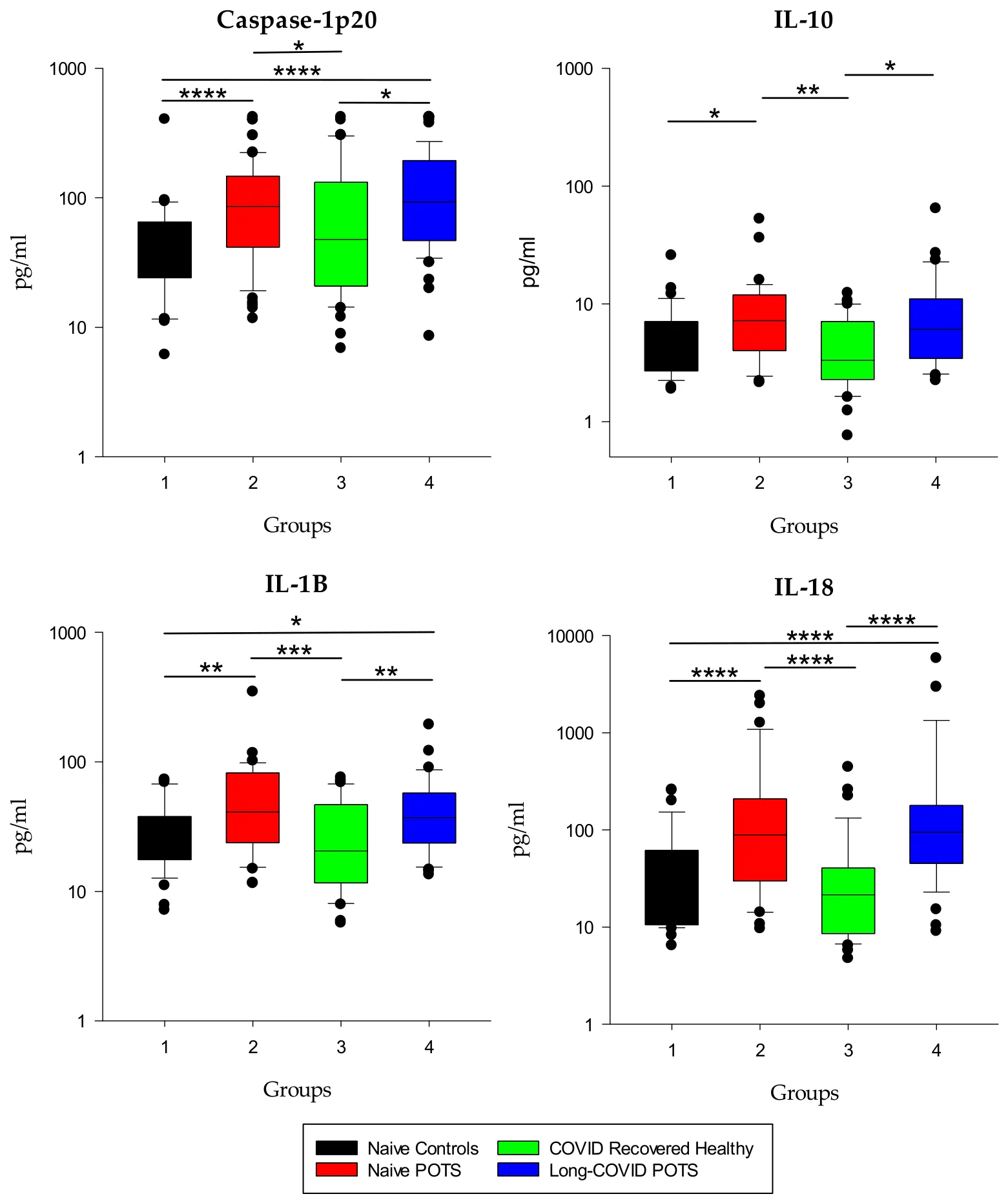
Innate immune marker concentrations across study cohorts. Box plots display the distribution of Caspase-1p20, IL-10, IL-18, and IL-1β in POTS, POTS COVID, and control cohorts. Horizontal brackets indicate significant pairwise differences between cohorts (adjusted post hoc significance: * *p* < 0.05, ** *p* < 0.01, *** *p* < 0.001, **** *p* < 0.0001).

**Figure 5:**
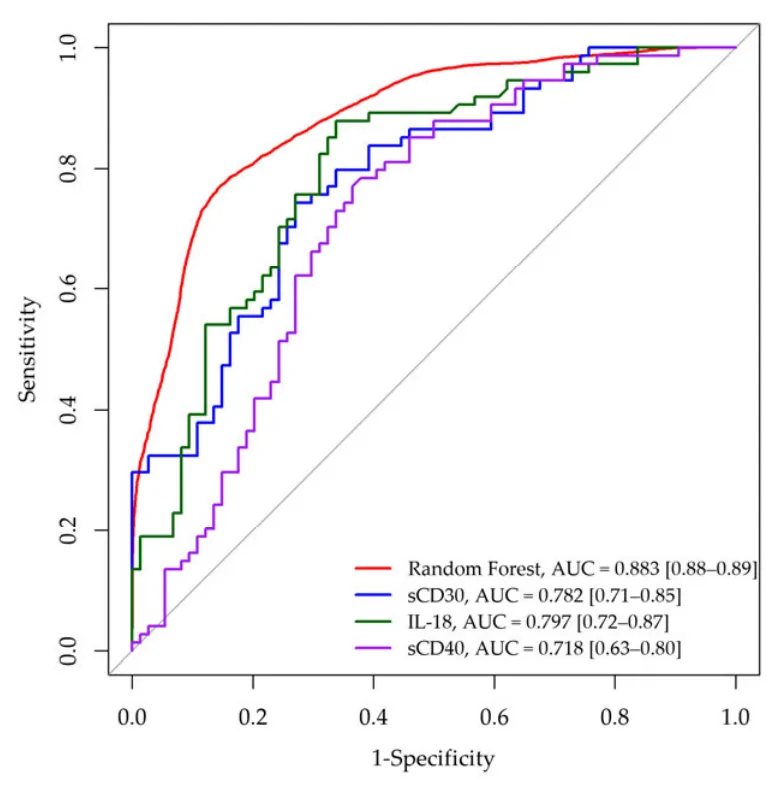
Receiver operating characteristic curves of the Random Forest model and individual biomarkers. ROC analysis illustrates that the combined Random Forest model achieves superior predictive accuracy for differentiating a combined POTS cohort from controls (AUC = 0.883 [0.88–0.89]). The combined model outperformed individual assessments of IL-18 (AUC = 0.797 [0.72–0.87]), sCD30 (AUC = 0.782 [0.71–0.85]), and sCD40 (AUC = 0.718 [0.63–0.80]).

Standardized mean differences (Cohen’s d) were calculated for the combined cohorts (Supplementary Table S3). sCD40L demonstrated the most robust discrimination (d = 0.82 [0.48, 1.15]), followed by sCD30 (0.74 [0.40, 1.07]). Medium-to-large effect sizes were also observed for key inflammatory markers Caspase1p20, IL-10, IL-1β, and IL-18 (Figure 4). The remaining cytokines exhibited moderate effect sizes (Supplementary Figure S1). Recursive feature elimination with random forest classification consistently selected eight features: sCD30, sCD40, IL-18, IL-21, IFN-γ, Caspase-1p20, sCD40L, and IL-1β. A random forest classifier constructed using this panel and potential confounders achieved excellent discriminatory power between POTS and control cohorts with a cross-validated AUC of 0.883 (out-of-bag error rate: 18.9%, Figure 5). Permutation-based feature importance identified IL-18 and sCD30 as the two most critical predictors whereas confounders of sex, age, and weight showed negligible importance (Supplementary Table S4). While the multivariate panel provided the highest accuracy, top individual cytokines remained predictive (sCD30 AUC = 0.782, IL-18 AUC = 0.787, and sCD40 AUC = 0.718).

To identify predictors of clinical severity within the POTS cohort, LASSO regression selected IL-17, Caspase1p20, and IL-18 from a 15-feature panel. A multivariate generalized linear model (GLM) adjusted for age and weight demonstrated that these three markers significantly predicted COMPASS-31 score (χ^2^ = 1.39, *p* = 0.015). In this model, IL-18 was a significant positive correlation; a two-fold elevation in IL-18 concentration was associated with a 5.6% increase in mean COMPASS-31 score (means ratio 1.06 [1.01, 1.11]). Conversely, IL-17 exhibited a significant inverse association with disease severity, where each two-fold increase corresponded to a 14.6% decrease in mean COMPASS-31 score (means ratio: 0.85, [0.78–0.94]). Caspase1p20, age, and weight were not statistically significant in the adjusted analysis.

## 4. Discussion

This investigation provides a comparative analysis of circulating innate immune profiles of patients with pre-pandemic POTS and Long COVID-associated POTS. Our principal findings reveal both POTS cohorts exhibit a broadly similar plasma cytokine signature compared to healthy controls, characterized by significant elevations of innate immune cytokines. Notably, IL-18 emerged as a key discriminative predictor. While our cross-sectional design cannot distinguish whether this systemic inflammation is an initiating trigger or a downstream consequence, these data demonstrate that pre-pandemic POTS and Long COVID-associated POTS share a congruent inflammatory phenotype among measured biomarkers. Demographically, our study groups are representative of the current literature of both Long COVID and POTS, exhibiting a marked female predominance (96%) alongside statistically equivalent age and weight distributions [35,36,37]. Crucially, autonomic symptom burden, as quantified by COMPASS-31 scores and clinical symptoms, shared no significant divergence between the cohorts. This clinical concordance provides the necessary context for comparison of their underlying immune signatures. Furthermore, to account for the established sexual dimorphism in cytokine expression [38,39], sensitivity and covariate adjusted analysis were performed, confirming that the observed biomarker pro-files remained robust following adjustment for sex.

Multivariate PERMANOVA analysis revealed no significant difference in the global cytokine profiles between the POTS and Long COVID POTS cohorts. This shared immunological signature was further validated by a random forest classifier, which achieved an AUC of 0.883 in distinguishing the combined POTS cohorts from controls. Importantly, be-cause this model relies on internal cross validation, subsequent evaluation in independent cohorts is necessary to establish true discriminative utility. These findings provide evidence of a common immune phenotype between POTS and Long COVID-associated POTS. Consequently, the immune dysregulation currently being characterized in Long COVID may provide a valuable framework for POTS [40,41,42]. However, further characterization is required to definitively establish if POTS is truly the result of an inciting immunological stressor.

The notable performance of IL-18 is supported by concurrent elevations of the active Capase-1 p20 subunit and IL-1β. Together, this triad of circulating biomarkers are suggestive of activation of canonical inflammasome pathways, most notably the NLRP3 inflammasome. While both IL-1β and IL-18 result from Caspase-1 proteolysis, the relative stability and longer half-life of IL-18 in plasma most likely contribute to its superior performance as an inflammatory biomarker [43].

A persistent inflammasome signature could reflect a non-resolving innate trigger, consistent with an established response to viral pathogen-associated molecular patterns (PAMPs) and endogenous damage-associated molecular patterns (DAMPs) through the TLR/NF-kβ pathway [44]. In the context of Long COVID, this inflammatory axis may be driven from several non-mutually exclusive mechanisms proposed for Long COVID pathogenesis including persistent viral reservoirs, intestinal dysbiosis with associated barrier permeability, mitochondrial dysfunction, and persistent micro-clots [45,46,47,48,49,50]. While the observed concentrations of IL-18, IL-1β, and Caspa-se-1p20 may be consistent with inflammasome signaling, our study did not directly measure intracellular assembly. Subsequent studies investigating NLRP3 expression, ASC speck formation, or intracellular Caspase-1 activity are required to confirm active inflammasome assembly.

The shared profile between POTS cohorts is characterized by an increase in innate immune cytokines (IL-1β, IL-18, IL-6, IL-8, TNFα), the regulatory mediator IL-10, markers of cellular activation (sCD30, sCD40, sCD40L) and an interferon-Th17 effector signature (IFNβ, IFNΥ, and IL-17). Elevations of IL-10 alongside pro-inflammatory cytokines may represent a complex, non-resolving immune state [51]. IL-10 has a pleiotropic role in inflammatory states; this pattern may additionally indicate T-cell exhaustion or a broader dysregulation of immune regulatory networks. Ultimately, these findings suggest a state of persistent inflammation with a lack of effective resolution by the immune system.

Persistent innate immune engagement is suggested by significant elevation in IL-6, IL-8, and TNFα [52,53]. Within the context of our data, these cytokines may represent a broader systemic amplification of the innate signal. While TNFα and IL-6 are classic mediators of systemic inflammation, IL-8 is notably an indicator of endothelial activation [54,55].

The concurrent elevations in sCD30, sCD40, and sCD40L suggest a potential connection between innate signaling and activation of adaptive immune pathways [56]. In this context, the release of sCD40L, largely derived from activated platelets, may contribute to endothelial activation and serve as a costimulatory signal for T-cells [57,58]. These findings remain associative, however, as circulating markers do not confirm functional recruitment of adaptive cells. Similarly, the observed IFNΥ and IL-17 signature is consistent with a Th1/Th17-skewed environment [59,60], though direct cellular phenotyping remains necessary to characterize lymphocyte subsets and their functional states. Interestingly, IL-21 was the sole cytokine that demonstrated no statistical divergence from controls. Stability in IL-21 signaling might suggest sustained effector T-cell signaling and innate-adaptive crosstalk rather than active B-cell recruitment.

Previous work has established a prevalence of delta-storage pool deficiency (δ-SPD) in POTS and Long COVID POTS [61,62,63]. Delta storage pool deficiency has both genetic and acquired etiologies; the current framework suggests that in POTS, an inflammatory state may be depleting the platelet into storage pool exhaustion. The platelet is now recognized as an integral innate immune cell which expresses Toll-like receptors 1–10, Major Histocompatibility Complex class I (MHC-I), and Fc ΥRIIA, stores sCD40L, IL-1β, and RANTES within alpha granules, and has been shown to produce IL-1β and IL-18 through expression of the NLRP3 inflammasome [64,65,66]. The thrombo-inflammatory environment reported in this study is consistent with identification of micro-clots and platelet–leukocyte aggregates in Long COVID cohorts [67,68].

While the observed immune profile provides a potential framework for interpreting the inflammatory features associated with POTS, the relationship between systemic cytokine concentrations and clinical manifestations appears complex. Although LASSO regression identified a subset of relevant features, the overall model accounted for only a modest proportion of variance of COMPASS-31 scores. Notably, IL-18 emerged as a positive correlation of symptom severity. The absence of strong global correlation is consistent with some clinical features of POTS—as a chronic, fluctuating disorder and a single measurement of circulating cytokines—that may not adequately reflect the cumulative inflammatory burden or the phenotypic variability driven by diverse non-immune factors.

This study is subject to several limitations that warrant consideration. The data represents a cross-sectional assessment of circulating biomarkers under a chronic condition, which may not fully capture the temporal dynamics of an inflammatory response. Consequently, these findings remain associative, and it is not possible to establish a definitive causal directionality. It is unclear whether these signatures represent causative drivers of the syndrome or are a secondary consequence. Additionally, the reliance on plasma biomarkers limits the ability to assess functional cellular activity or tissue-specific responses. The POTS and POTS Long COVID cohorts are predominantly female. While this reflects the established epidemiology of POTS and Long COVID, it limits the generalization of these findings. Although sex-based immunologic dimorphism may contribute to variance in cytokine signaling, sex did not emerge as a significant predictive covariate in the Random Forest model. Furthermore, the absence of patients with alternative triggers to POTS, such as concussion, surgery, growth spurt, menarche, or immunization, constrains our ability to determine if the observed immune patterns are universal across all phenotypes of POTS. Further longitudinal research in larger cohorts is needed to validate these observations.

## 5. Conclusions

This investigation highlights a distinct, shared cytokine profile in POTS and Long COVID-associated POTS, consistent with an active innate immune system. These findings suggest an immunological relationship between primary viral insult and chronic dysautonomia, suggesting that clinical successes in Long COVID research may be applicable to the broader POTS population. Future investigation is warranted to explore the potential role of NLRP3-related platelet activation, dysregulated immune pathways, and lymphocyte immunophenotypes in the immune landscape of POTS and Long COVID.

## Figures and Tables

**Table 1:** Study subject demographics.

	COVID Naïve	POTS	COVID-Recovered	POTS COVID	*p*-Value ^1^
**Age**	31.2 (11.7)	34.1 (13.8)	36.5 (13.5)	39.3 (14.9)	0.3
**Sex (F:M)**	62%	86%	77%	96%	0.002
**Weight (lbs)**	163.9 (52.6)	161.2 (46.2)	172.6 (48.7)	161.8 (37.1)	0.6
**COMPASS-31 score**	3.0 (9.3)	36.0 (17.0)	8.0 (8.0)	34.0 (15.0)	<0.001

^1^ One-way analysis of means; Pearson’s Chi-squared test; Kruskal–Wallis rank sum test.

**Table 2:** Univariate cytokine differences.

	COVID Naïve ^1^	POTS ^1^	COVID-Recovered ^1^	POTS COVID ^1^	*p*-Value ^2^	Adjusted *p*-Value ^3^
**IL-1β**	21.6 (18.1–37.1)	40.9 (24.5–79.2)	20.5 (11.7–46.1)	37.1 (24.6–56.8)	<0.001	<0.001
**IL-6**	12.0 (7.5–20.3)	17.3 (9.5–30.9)	9.4 (5.1–21.3)	19.7 (10.5–23.8)	0.020	0.023
**IL-8**	14.0 (8.5–30.2)	21.6 (12.8–37.5)	10.1 (6.6–24.3)	21.6 (13.8–34.8)	0.003	0.005
**IL-10**	4.0 (2.7–7.0)	7.2 (4.1–11.9)	3.3 (2.4–7.0)	6.8 (3.5–11.3)	0.002	0.004
**IL-17**	10.2 (7.4–17.3)	18.0 (9.9–28.2)	10.8 (6.7–15.3)	12.8 (8.8–21.1)	0.003	0.006
**IL-18**	18.8 (10.5–60.0)	88.9 (30.2–195.9)	21.4 (8.6–37.1)	94.9 (50.5–165.9)	<0.001	<0.001
**IL-21**	100.0 (67.0–154.9)	136.0 (71.0–223.1)	78.8 (57.5–161.2)	106.7 (71.5–172.0)	0.14	0.14
**sCD30**	117.7 (92.4–168.9)	301.3 (179.3–463.0)	119.5 (54.1–228.2)	254.6 (133.9–421.4)	<0.001	<0.001
**sCD40**	183.3 (131.5–290.1)	308.0 (239.5–445.2)	149.9 (97.7–321.4)	272.7 (187.9–432.2)	<0.001	<0.001
**sCD40L**	717.9 (604.0–964.7)	1184.3 (822.1–1676.0)	747.3 (545.3–1026.2)	1070.8 (901.3–1360.3)	<0.001	<0.001
**IFNβ**	1514.2 (1192.1–2335.5)	2668.8 (1604.9–3824.7)	1487.1 (899.8–2576.7)	2432.5 (1633.6–3663.4)	0.005	0.007
**IFNΥ**	3.8 (2.6–7.6)	7.3 (5.5–12.8)	3.1 (1.5–6.6)	7.8 (4.1–13.3)	<0.001	<0.001
**MCP-1**	695.7 (436.7–971.0)	910.3 (471.7–1351.2)	577.7 (343.8–954.1)	766.6 (565.2–1073.9)	0.040	0.043
**TNFα**	1.4 (0.1–10.7)	12.5 (0.7–35.8)	0.6 (0.0–6.7)	7.3 (1.3–26.9)	0.008	0.010
**Caspase-1p20**	32.3 (16.8–62.1)	84.8 (43.3–126.0)	47.0 (21.1–104.0)	86.4 (46.6–194.5)	<0.001	<0.001

^1^ Median (Q1–Q3); ^2^ Kruskal–Wallis rank sum test; ^3^ Benjamini and Hochberg correction for multiple testing (false discovery rate threshold of 0.05 across 15 comparisons).

## Data Availability

The original contributions presented in this study are included in the article/Supplementary Materials. Further inquiries can be directed to the corresponding authors.

## Supplementary Materials

The following supporting information can be downloaded at: https://www.mdpi.com/article/10.3390/biomedicines14071605/s1, Figure S1: Univariate biomarker differences between study groups; Table S1: Female-only sensitivity analysis of biomarkers; Table S2: Multiple Linear Regression Coefficients Confounding For Sex; Table S3: Biomarker Effect Sizes; Table S4: Random Forest Feature Performance.

## Abbreviations

The following abbreviations are used in this manuscript:

ARDS	Acute respiratory distress syndrome
ASC	Apoptosis-associated speck-like protein containing a CARD
AUC	Area under the curve
BPM	Beats per minute
CARD	Caspase recruitment domain
CD	Cluster of differentiation
COMPASS-31	Composite Autonomic Symptom Score-31
DAMP	Damage-associated molecular pattern
ELISA	Enzyme-linked immunosorbent assay
GLM	Generalized linear model
HPV	Human papilloma virus
IFN	Interferon
IL	Interleukin
LASSO	Least Absolute Shrinkage and Selection Operator
MHC	Major histocompatibility complex
NLRP3	NOD-like receptor protein 3
NOD	Nucleotide-binding oligomerization domain
PAMP	Pathogen-associated molecular pattern
PERMANOVA	Permutational multivariate analysis of variance
POTS	Postural orthostatic tachycardia syndrome
RANTES	Regulated upon Activation, Normal T-cell Expressed and Secreted
RFE	Recursive feature elimination
SE	Standard error
SPD	Storage pool deficiency
Th	T helper cell
TLR	Toll like receptor
TNF	Tumor necrosis factor
