# Supplementary material for: Plasma Cytokine and Caspase-1p20 Profiles in Pre-Pandemic and Long COVID-Associated Postural Orthostatic Tachycardia Syndrome
Source: Biomedicines. 2026 Jul 17;14(7):1605. doi: 10.3390/biomedicines14071605 (PMC13406494; doi:10.3390/biomedicines14071605)

Supplemental Figure S1: Univariate biomarker differences between study groups

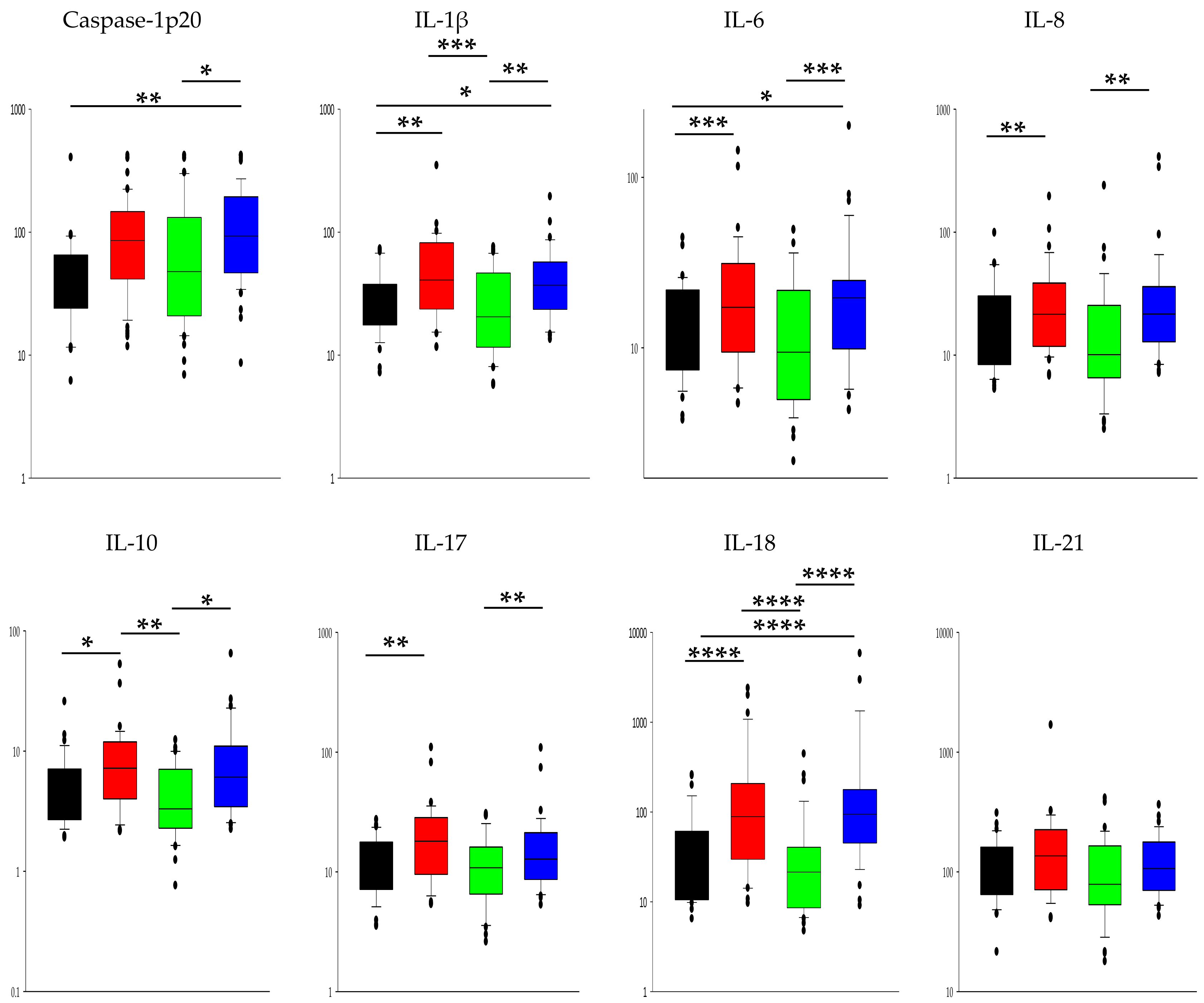

Supplemental Figure S1: Univariate biomarker differences between study groups

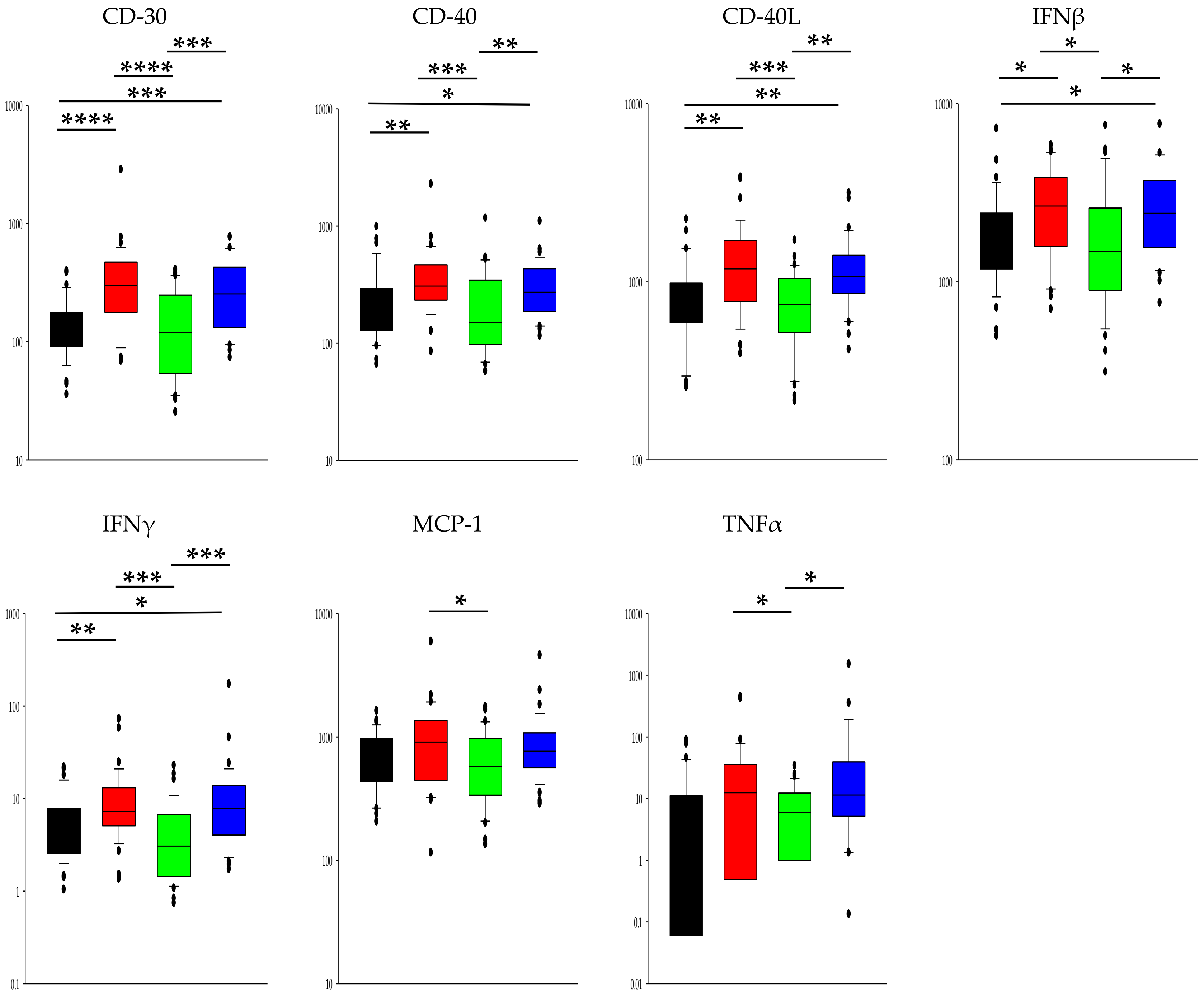

Supplement: Supplementary file 1 [file biomedicines-14-01605-s001.zip › Supplemental Figure S1 of Cytokines 7.08.26.pdf]
