# Supplementary material for: Plasma Cytokine and Caspase-1p20 Profiles in Pre-Pandemic and Long COVID-Associated Postural Orthostatic Tachycardia Syndrome
Source: Biomedicines. 2026 Jul 17;14(7):1605. doi: 10.3390/biomedicines14071605 (PMC13406494; doi:10.3390/biomedicines14071605)
Supplement: Supplementary file 1 [file biomedicines-14-01605-s001.zip › Supplemental Table S1.pdf]

Supplemental Table S1. Female-only sensitivity analysis of biomarkers.

	Control	POTS	p-value[2]	q-value[3]
**IL-1β**	22.1 (15.6 - 46.1)	47.2 (26.7 - 70.9)	<0.001	<0.001
**IL-6**	12.1 (8.1 - 23.4)	19.7 (10.7 - 28.4)	0.028	0.031
**IL-8**	12.9 (8.1 - 28.8)	22.9 (13.8 - 37.5)	0.009	0.011
**IL-10**	4.7 (2.7 - 7.2)	7.3 (4.2 - 11.7)	0.003	0.004
**IL-17**	10.9 (7.4 - 17.0)	15.2 (10.7 - 23.9)	0.011	0.012
**IL-18**	14.7 (10.1 - 37.1)	100.0 (39.7 - 207.7)	<0.001	<0.001
**IL-21**	110.3 (58.0 - 181.4)	134.6 (73.5 - 190.7)	0.15	0.15
**sCD30**	123.3 (69.0 - 241.8)	297.8 (179.1 - 442.3)	<0.001	<0.001
**sCD40**	183.3 (122.9 - 321.4)	312.7 (223.5 - 442.2)	<0.001	<0.001
**sCD40L**	805.4 (631.5 - 1,100.1)	1,120.5 (901.3 - 1,542.4)	<0.001	<0.001
**Caspase-1p20**	37.2 (14.1 - 57.0)	87.6 (45.6- 150.7)	<0.001	<0.001
**IFN-β**	1,537.6 (944.6 - 2,626.2)	2,522.0 (1,716.9 - 3,859.5)	0.002	0.003
**IFN-γ**	4.5 (2.4 - 8.3)	8.7 (4.3 - 13.3)	<0.001	0.001
**MCP-1**	654.1 (370.3 - 969.5)	849.8 (565.2 - 1,301.6)	0.005	0.007
**TNFα**	5.1 (0.5 - 13.1)	13.3 (5.2 - 36.1)	0.001	0.003

[1]Median (Q1 - Q3) [2]Kruskal-Wallis rank sum test [3]Benjamini & Hochberg correction for multiple testing
