# Supplementary material for: Plasma Cytokine and Caspase-1p20 Profiles in Pre-Pandemic and Long COVID-Associated Postural Orthostatic Tachycardia Syndrome
Source: Biomedicines. 2026 Jul 17;14(7):1605. doi: 10.3390/biomedicines14071605 (PMC13406494; doi:10.3390/biomedicines14071605)
Supplement: Supplementary file 1 [file biomedicines-14-01605-s001.zip › Supplemental Table S2.pdf]

Supplemental Table S2. Multiple Linear Regression Coefficients Confounding For Sex.

	**Estimate [95% CI]**
IL-1β	0.509 [0.270, 0.747]
IL-6	0.309 [0.048, 0.570]
IL-8	0.409 [0.111, 0.706]
IL-10	0.412 [0.167, 0.657]
IL-17	0.353 [0.143, 0.564]
IL-18	1.579 [1.125, 2.033]
IL-21	0.189 [-0.046, 0.425]
sCD30	0.733 [0.479, 0.987]
sCD40	0.464 [0.242, 0.687]
sCD40L	0.407 [0.235, 0.580]
Caspase-1p20	0.744 [0.362, 1.127]
IFN-γ	0.524 [0.260, 0.788]
MCP-1	0.353 [0.128, 0.577]
TNF-α	0.864 [0.301, 1.427]
