# Supplementary material for: Plasma Cytokine and Caspase-1p20 Profiles in Pre-Pandemic and Long COVID-Associated Postural Orthostatic Tachycardia Syndrome
Source: Biomedicines. 2026 Jul 17;14(7):1605. doi: 10.3390/biomedicines14071605 (PMC13406494; doi:10.3390/biomedicines14071605)
Supplement: Supplementary file 1 [file biomedicines-14-01605-s001.zip › Supplemental Table S3.pdf]

Supplemental Table S3. Biomarker Effect Sizes.

	**Cohen's *d* [95% CI]**
sCD40L	0.82 [0.48, 1.15]
sCD30	0.74 [0.40, 1.07]
IL-1β	0.65 [0.32, 0.98]
IL-17	0.58 [0.25, 0.90]
IL-18	0.50 [0.17, 0.83]
Caspase-1p20	0.50 [0.19, 0.80]
CD40	0.48 [0.15, 0.81]
IL-6	0.47 [0.14, 0.80]
IFN-β	0.46 [0.13, 0.79]
IFN-γ	0.46 [0.13, 0.79]
IL-8	0.32 [0.01, 0.64]
IL-21	0.29 [0.04, 0.61]
IL-10	0.28 [0.05, 0.60]
