# Supplementary material for: Plasma Cytokine and Caspase-1p20 Profiles in Pre-Pandemic and Long COVID-Associated Postural Orthostatic Tachycardia Syndrome
Source: Biomedicines. 2026 Jul 17;14(7):1605. doi: 10.3390/biomedicines14071605 (PMC13406494; doi:10.3390/biomedicines14071605)
Supplement: Supplementary file 1 [file biomedicines-14-01605-s001.zip › Supplemental Table S4.pdf]

Supplemental Table S4. Random Forest Feature Performance

Feature	Mean Decrease Accuracy	Mean Decrease Gini
IL-18	40.70	18.23
CD30	32.73	12.60
Age	17.71	7.29
CD40	15.64	5.99
IL-21	13.50	6.99
CD40L	13.02	4.14
IFN-γ	11.70	5.15
Caspase-1p20	11.56	5.66
MCP-1	3.01	3.87
Weight	-1.70	3.55
